# Phenolic Compounds in Whole Grain Sorghum and Their Health Benefits

**DOI:** 10.3390/foods10081921

**Published:** 2021-08-19

**Authors:** Jingwen Xu, Weiqun Wang, Yong Zhao

**Affiliations:** 1 College of Food Science and Technology, Shanghai Ocean University, Shanghai 201306, China; yzhao@shou.edu.cn; 2 Department of Food Nutrition Dietetics and Health, Kansas State University, Manhattan, KS 66506, USA; wwang@ksu.edu

**Keywords:** sorghum phenolics, antioxidant, anti-inflammatory, anti-proliferative, anti-diabetic, anti-atherogenic

## Abstract

Sorghum grain (*Sorghum bicolor* L. Moench) is a staple food grown across the globe, and is mainly cultivated in the semi-arid regions of Africa and Asia. Recently, sorghum grain is increasingly utilized for human consumption, due to the gluten-free nature and potential phenolic-induced health benefits. Sorghum grain is rich in bioactive phenolic compounds, such as ferulic acid, gallic acid, vanillic acid, luteolin, and apigenin, 3-deoxyanthocyanidins (3-DXA), which are known to provide many health benefits, including antioxidant, anti-inflammatory, anti-proliferative, anti-diabetic, and anti-atherogenic activities. Given an increasing trend of sorghum consumption for humans, this article reviews the content and profile of phenolics in sorghum. It covers aspects of their health benefits and explores their mechanisms of action. The impact of thermal processing, such as boiling, steaming, roasting, and extrusion on sorghum phenolics is also discussed. Compelling data suggest the biological functions of sorghum phenolics, however, further investigations appear warrant to clarify the gap in the current research, and identify promising research topics in future.

## 1. Introduction

Sorghum (*Sorghum bicolor* L. Moench) is the fifth most produced cereal crop globally after wheat, maize, rice, and barley, and is the main cereal food consumed in the semi-arid regions of Africa and Asia, due to the high resistance to drought [1]. Sorghum grain possesses tannins, which are bitter tasting. Sorghum grain used to be utilized for animal feed and biofuel production, rather than human food in the United States. However, sorghum is the main cereal grain for the populations in sub-Saharan Africa. In the last decade, the US has selected less tannin variants, and there has been a growing interest of sorghum consumption due to the gluten-free nature. Celiac disease is an immune disorder which affects millions of people in the US, which results from the consumption of glutamine-rich cereal grains, such as wheat, barley, and rye. The gluten-free nature of sorghum shows its great potential as an alternative cereal grain for human consumption by eliminating the risk of celiac disease for celiac patients. Except for the gluten-free nature, sorghum grain possesses bioactive phenolic compounds, such as phenolic acids, flavonoids, and anthocyanins [2,3], which are known to be associated with reducing the risk of many chronic diseases, such as diabetes, obesity, cancer, and cardiovascular disease [4,5,6,7]. More studies have recently been focused on the processing and health-promoting effects of sorghum phenolics.

Sorghum grain is composed of pericarp, testa, endosperm, and germ from the outside to the inner. Testa is located between the pericarp and endosperm, which is unique in the sorghum grains and distinct from other cereal grains [8]. Sorghum grain contains pigmented pericarp (i.e., black, red, yellow, brown) and non-pigmented pericarp (i.e., white) [9]. According to Dykes et al. [10], the genes R and Y contribute to the pericarp color, for example, a white color is shown when Y is homozygous recessive; a yellow color is shown when R and Y are homozygous recessive and homozygous dominant; and a red color is shown when R and Y are dominant. Bioactive phenolic compounds are primarily located in the pericarp and testa, wherein they are bound to the non-starch polysaccharides, such as cellulose, hemicellulose, lignin, and pectin in cell wall [11]. Dykes et al. [12] demonstrated that factors, such as varieties and growing conditions, determined the content and the profile of phenolic compounds in sorghum grains. Generally, pigmented sorghum grains contain more content of phenolics than the white sorghum grains, due to the presence of pigmented anthocyanins [3,13,14,15].

Previously, review articles have been focused on the bioactive compounds in sorghum and their health benefits as potential food ingredients [16], and the processing technologies for reducing anti-nutritional factors in sorghum grain [17]. To date, there is no comprehensive review regarding the phenolics in sorghum grain and their biological functions, such as antioxidant activity, anti-inflammation activity, anti-cancer effect, anti-diabetic, and anti-atherogenic functions. Given the increasing consumption of sorghum phenolics, this review article will focus on the health-promoting effect of phenolic compounds in sorghum, with regards to the recent antioxidant activity, anti-inflammation activity, anti-proliferative effect, anti-diabetic, and anti-atherogenic functions, aimed to better understand the biological functions of phenolics in sorghum grains for improving human health. The effect of thermal processing on phenolics in sorghum grains, in terms of content and profile, will also be discussed. The current research gap will be identified, and promising research topics will be further recommended.

## 2. Phenolic Compounds in Sorghum Grain

**Figure 1:**
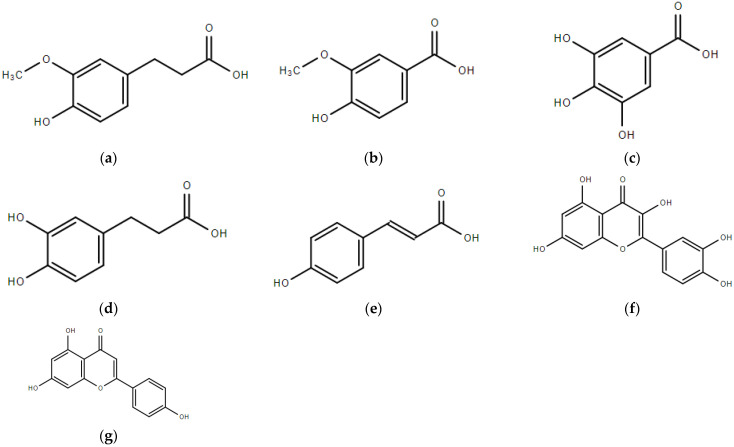
Typical phenolics in sorghum grains, (**a**) ferulic acid; (**b**) vanillic acid; (**c**) gallic acid; (**d**) caffeic acid; (**e**) *p*-coumaric acid; (**f**) luteolin; (**g**) apigenin.

Phenolic compounds, belonging to secondary metabolites, are well-known to naturally bio-synthesize in plants. Sorghum grain possesses many phenolic acids and flavonoids, wherein flavonoids can be further divided into flavonone, flavonol, anthocyanins, and condensed tannins, known as proanthocyanidins in sorghum. Caffeic acid, cinnamic acid, ferulic acid, gallic acid, salicylic acid, vanillic acid, and *p*-coumaric acid dominate the phenolic acids in sorghum grains (shown in Figure 1) [18,19]. The predominant flavonoids in sorghum include luteolin, apigenin, eriodictyol, and naringenin [12]. We previously reported that 3-deoxyanthocyanidins (3-DXA) was the predominant anthocyanins in sorghum grains, which mainly included luteolinidin, apigeninidin, 5-methoxyluteolinidin, and 7-methoxy apigeninidin. This was found through the identification of anthocyanins in 25 sorghum grains with various pigmented pericarps, including red, brown, yellow, and white pericarps [13]. Except for the predominant phenolic acid and flavonoids, stilbenoids and polyamines are also present in sorghum grains in a small amount. The content of silbenoid trans-piceid and trans-resveratrol of red sorghum is reported to be 0.4–1.0 mg/kg and 0.2 mg/kg [20]. Total phenolic content, total flavonoid content, and total anthocyanin content of sorghum varieties associated with pigmented pericarp sorghum grains are summarized in Table 1.

Conventionally, phenolic compounds in sorghum grain are mainly obtained through refluxing extraction, water extraction, maceration extraction, soxhlet extraction, and organic solvent extraction [3,12,17,19]. However, the extraction yield, content, and profile of phenolics in sorghum are varied between the different extraction solvents. For example, Devi et al. [23] reported that the acidified methanol extract of sorghum (red sorghum, collected in Tamil Nadu, India) bran polyphenols showed greater content of anthocyanins (4.7 mg/g) than methanol extract (1.95 mg/g) and acetone extract (1 mg/g). To the content of total flavonoids and phenolics of sorghum bran, acidified methanol extract was also higher than methanol extract and acetone extract.

Nowadays, emerging technologies have been utilized for phenolics extraction from sorghum grain, aimed to improve the extraction yield and phenolics content. So far, ultrasound-assisted extraction [24], pulsed-electric field, accelerated solvent extraction [25], microwave-assisted extraction [26], and subcritical water extraction [27] have been reported for phenolic extraction. For example, the accelerated solvent extraction method at a temperature of 120 and 150 °C, by using solvent of 50% and 70% ethanol/water (*v*/*v*), could result in the content of phenolic compounds of black sorghum bran (A05028/RTx3362), which is up to 45 mg/g gallic acid equivalent of dry weight (gallic acid equivalent, dry weight) (GAE, dw) [25]. Luo et al. [24] reported that the polyphenolic content of red sorghum bran was 49.7 mg/g GAE (dw), through an ultrasound-assisted extraction method for 21 min of processing time, 53% ethanol of solvent, and 52:1 mL/g of solid–liquid ratio. Luo et al. [27] developed the subcritical water extraction method for extracting phenolics up to 47.2 mg/g GAE (dw) from sorghum, through optimized conditions, including 144.5 °C of temperature, 21 min of extraction, and 35 mL/g of solid–liquid ratio.

The identification, characterization, quantification, and qualification of phenolic compounds from sorghum rely on chromatographic techniques, such as high performance liquid chromatography (HPLC) combined with different detectors, including diode array detector (DAD) [13,27], UV-vis photodiode array detector (PDA) [21], tandem quadrupole detector mass spectroscopy (TQD-MS) [26], electron spray ionization (ESI), and atmospheric pressure chemical ionization (APCI) triple quadrupole MS [3,28].

## 3. Health Benefits and Potential Molecular Mechanisms of Phenolic Compounds in Sorghum Grain

### 3.1. Antioxidant Activity

Overproduction of reactive oxygen species (ROS) in the human body will potentially result in oxidative stress, which is implicated in the increasing risk of many chronic diseases, such as inflammation, diabetes, atherosclerosis, and cardiovascular disease [29]. Antioxidants from dietary polyphenols can scavenge free radicals for preventing chronic diseases [29]. This section will mainly focus on the antioxidant activity of phenolics isolated from sorghum grains through tests from both in vitro and in vivo.

Phenolic compounds from sorghum varieties have been reported to possess antioxidant activities, which are mainly characterized by scavenging the radicals of DPPH, ABTS, FRAP, and ORAC in vitro [2,15,30]. For example, the ORAC value of phenolics isolated from black sorghum (Shawaya) bran was 3.7 mmol Trolox equivalents/mg (TE/mg) [31]. The IC_50_ value of DPPH radical scavenging activity of eight brown sorghum genotypes (SOR 01, SOR 03, SOR 08, SOR 11, SOR 17, SOR 21, SOR 24, SOR 33) varied from 91.2 to 361.2 mg/mL, and the IC_50_ value of ABTS radical scavenging activity ranged from 203.4 to 352.6 mg/mL [28]. Brown pericarp sorghum (IS131C) was found to possess greater antioxidant activity than black sorghum (Shawya Short Black 1), red sorghum (Mr-Buster, Cracka), and white sorghum (Liberty) when compared the antioxidant activity assayed by ABTS, DPPH, and FRAP [15]. According to Xiong et al. (2021), varieties of IS31C and Shawya Short Black 1 showed higher values of ABTS, DPPH, and FRAP than varieties of Liberty, Mr-Buster, and Cracka [15].

3-DXA in sorghum has also been shown to reduce the oxidative stress in vitro, through modulating the defense system against oxidative stress and inducing NADH: quinone oxyreductase (NQO) activity [4,31]. Belonging to the phase II enzyme, NAD(P)H quinone reductase is known as a detoxifying enzyme, balancing the carcinogen-activating phase I enzymes. The 2−3 double bond in the C-ring of flavonoids and 3-deoxyflavonoids in sorghum is considered to induce NQO activity [4].

Human colorectal cancer Caco-2 cells and hepatocarcinoma HepG2 cells are two common cellular antioxidant activity testing models through MTT assay [7,15]. EC_50_ values of sorghum varieties (i.e., Liberty, Mr-Buster, Cracka, IS131C, Shawaya Short Black 1) varied from 0.4 to 127.1 mg/mL, which assessed the total antioxidant effect of both extracellular and intracellular environments [15]. The median effective concentration (EC_50_) is the concentration of substance in an environmental medium expected to produce an effect in 50% of test organisms.

The antioxidant activity of sorghum phenolics in vivo has also been investigated through enzymatic activity, such as SOD and GPx. Lewis [32] studied the effect of a diet containing fiber from black sorghum (containing 3-DXA) and white sorghum (containing phenolic acids) on cellular antioxidant activity in rats. Black sorghum (3-DXA rich sorghum) resulted in an increase in superoxide dismutase activity (SOD) and a decrease in glutathione peroxidase (GPx) activity in normolipidemic rats [32]. SOD is a cellular antioxidant enzyme, which can catalyze the dismutation of superoxide anion to hydrogen peroxide, thus detoxifying oxygen and water by catalase or GPx [33]. GPx is an important antioxidant enzyme for reducing hydrogen and lipid peroxides. In addition, white sorghum (containing phenolic acids) increases the catalase activity (CAT) [32]. Catalase is known as a cellular hydrogen peroxide scavenger. Taken together, black sorghum (containing 3-DXA) and white sorghum (containing phenolic acids) show strong antioxidant activity in the rat model. However, Moraes et al. [34] reported diets containing sorghum flour (i.e., BRS 305, BRS 309, BRS 310) did not significantly influence the SOD level in normolipidemic rats. In another study conducted by Ajiboye et al. [35], phenolic extracts of red sorghum variety (obtained from Igbona market, Osogbo, Nigeria, 100 mg/kg body weight) increased the detoxifying enzymes in ROS, including SOD, CAT, GPx, glutathione reductase (GSH-Red), and glucose 6-phosphate dehydrogenase (*Glc* 6-PD) in rat liver. Taken together, the antioxidant activity of sorghum phenolic extracts in vivo is highly variable, and is dependent on the sorghum varieties, sorghum bran, sorghum flour, and whole grain of sorghum. Sorghum bran phenolic extracts showed greater antioxidant activity than sorghum flour, due to the presence of a higher content of phenolics in the bran. The antioxidant activity of sorghum phenolic extracts is summarized in Table 2.

### 3.2. Anti-Inflammatory Effect

Inflammation refers to an immune response to cellular injury or infection by pathogens, and triggers many chronic diseases. Pro-inflammatory cytokines, such as interleukin 1, β (IL-1β), tumor necrosis factor (TNF-α), and interleukin 6 (IL-6) are known to be involved in the inflammation pathogenesis through various cellular and molecular pathways. Phytochemicals are reported to modulate inflammation by inhibiting pro-inflammatory enzymes [36,37]. Therefore, this section will focus on the effect of phenolic extracts from sorghum grains on inhibiting inflammation in vitro and in vivo.

Black sorghum bran phenolics extract (10% *w*/*v* in 50% ethanol) showed an inhibitory effect on TNF-α and IL-1β in lipopolysaccharide-stimulated peripheral blood mononuclear cells at dilutions of 1:100–1:200 and 1:100–1:400, respectively [38]. Hong et al. [39] showed that acidified ethanol extracts of sorghum (SC84MX, SC84KS, PI570481) at 50 mg gallic acid equivalent/mL inhibited nitric oxide (NO) production up to 72.45%, 68.32%, and 95.36%, respectively. The increase in the secretion of TNF-α and IL-6 of RAW 264.7 macrophages infected by the bacteria *Legionella pneumophila* was observed after the treatment of polyphenol extracts of sorghum (PI570481) (0.625 and 1.25 mg/mL) [39]. The mRNA expressions of IL-6 and IL-β of RAW 264.7 macrophage cells were significantly inhibited by the soluble phenolic extracts of the sorghum variety (Tong Za 117) at 300 to 500 mg/mL and 50 to 500 mg/mL, respectively, though a non-toxic mechanism [37].

In addition to the in vitro evaluation, the inhibitory effect of sorghum phenolic extracts on inflammation is also reported in vivo. Black sorghum bran phenolic extracts also showed an anti-inflammatory effect on an 12-*O*-tetradecanoylphorbol acetate (TPA) induced mouse ear model [38]. In addition, golden gelatinous sorghum extracts inhibited the expression levels of cyclooxygenase-2 and inducible nitric oxide synthase, through a TPA induced mice ear edema model [36]. Ritchie et al. [40] studied the inhibitory effect of diets containing 6% dietary fiber from sorghum brans, including black bran (high levels of 3-DXA), Sumac bran (high levels of condensed tannins and low levels of 3-DXA), and a combination of high-tannin bran and black bran on colon inflammation. Diets containing sorghum bran upregulated the colonocyte proliferation and gene expression of trefoil factor (Tff3), and transformed growth factor beta (Tgfb) after the inflammation induced by DSS [40]. Tff3 and Tgfb are known to repair lesions and maintain epithelial barrier integrity, which are both involved in cellular migration and suppression of apoptosis. The effect of extruded sorghum flour on inflammation and oxidative stress in high fat diet-fed rats was studied by de Sousa et al. [41]. A diet containing extruded sorghum flour increased the total antioxidant capacity of serum plasma and SOD level, but reduced the concentrations of p65 through NF-κB in liver and lipids peroxidation [41]. The anti-inflammatory effect of sorghum phenolic extracts is summarized in Table 3.

So far, most studies have shown the anti-inflammatory effect of sorghum phenolic extracts. However, the underlying mechanism is not fully understood. In addition, studies have focused on the anti-inflammatory effect of phenolic extracts of sorghum grains, rather than the individual phenolic compounds. Therefore, little is known about which phenolic compound dominates to inhibit inflammation. Taken together, more studies are still warranted for further investigation regarding the the inhibitory effect of sorghum phenolics on inflammation.

### 3.3. Anti-Proliferative Effect

Cancer is a complex disease, involving the functioning oncogenes, de-functioning tumor suppressor genes, and tumor mutations caused by the endogenous and exogenous factors [45]. This section will focus on the effect of phenolic extracts from sorghum grains on cancer inhibition in vitro and in vivo.

Studies have reported sorghum phenolic extracts possessthe antioxidant activity, phase II enzyme induction, regulation of p53 gene, anti-proliferative effect on cancer cells, and induction of cancer cell apoptosis [4,7,46,47,48]. Quercetin, a flavonoid found in sorghum grain, has been reported to inhibit b-catenin signaling in SW480 colon cancer cells [49]. Luteolin, a predominant flavonoid in sorghum has also shown an anti-proliferative effect on human colorectal cancer HCT15 and CO115 cells, by harboring KRAS and BRAF activating mutations [50].

The IC_50_ values obtained through an MTT test of sorghum (i.e., KARI-Mtama, Mizzou, Tx430, Seredo, Sumac, Hi-tannin) phenolic extracts inhibiting HT-29 and OE33 cancer cell proliferation ranged from 54.8 to 389 mg/mL and 95.3 to 654 mg/mL, respectively [4]. Black sorghum 3-DXA extract has also been shown to have an inhibitory effect on HT-29 human colon cancer cells (IC_50_ = 180−557 mg/mL) [46]. Hargrove et al. [51] reported that phenolic extracts from sorghum (Sumac sorghum, black sorghum) bran inhibited the aromatase activity in vitro, which had IC_50_ values of 12.1 and 18.8 mg/mL, respectively. Suganyadevi et al. [52] reported that 3-deoxyanthocyanins in red sorghum bran induced apoptosis in breast cancer MCF 7 cells through stimulating the p53 gene and down-regulating the Bcl-2 gene. P53 gene is known to be responsible for the cell cycle arrest and apoptosis. Phenolic extracts of black pericarp sorghum have also been shown to inhibit the growth of human HepG2 cells and Caco-2 cells, through a cell cycle arrest at G2/M phase and an induction of apoptosis [7].

In addition to the sorghum bran, sorghum stalk phenolic extract has also been shown to have an inhibitory effect against colon cancer proliferation in vitro. Massey et al. [53] isolated the phenolics from the pith and dermal layer of sweet sorghum (i.e., Dale and M81E) stalk, and found that the dermal layer contained more content of phenolics than the pith for both varieties, especially the content of 3-DXA apigeninidin and luteolinidin. Phenolic extracts from the dermal layer of sorghum varieties Dale and M81E showed higher antioxidant activity than the pith assayed by ABTS [53]. The extract of dermal layer of sweet sorghum Dale inhibited the growth of colon cancer HCT116 cells and colon cancer stem cells (CCSCs), through modulating the gene p53 above 35 mg of gallic acid equivalent/mL [53].

The inhibitory effect of sorghum phenolic extracts on cancer has also been reported. Wu et al. [54] found an antioxidant activity of sorghum (Moench) procyanidins (150 mg/kg) against oxidative stress in a rat model, that significantly reversed the increase in malondialdehyde (MDA) level and decreased SOD and GPx in both liver homogenate and serum of rat induced by D-galactose. Sorghum procyanidins (100, 200, 400 mg/kg) inhibited the tumor growth and reduced tumor weight in C57BL/6J mice of lung cancer, and the inhibitory effect of tumor growth and weight was dose-dependent, through the suppression of vascular endothelial growth factor (VEGF) production in mice [54]. Hwanggeumchal sorghum phenolic extracts have also been shown to have an inhibitory effect on human breast cancer MDA-MB-231 cells and MC7 xenografts in mice, through modulating Jak/STAT pathways, hindering the STAT5b/IGF-1R and STAT3/VEGF pathways, and down-regulating the angiogenic factors, such as VEGF, VEGF-R2, and cell cycle regulators such as cyclin D, cyclin E, and pRb [55]. In addition, Hwanggeumchal sorghum extracts also induced the apoptosis of MDA-MB-231 cells arrested at G1 phase. The anti-proliferative effect of sorghum phenolic extracts is summarized in Table 4.

To date, the anti-proliferative effect of sorghum phenolic extracts has been investigated in regards to the effective dose and underlying mechanism. However, most studies have been focused on the complex phenolic extracts of sorghum, rather than the individual phenolic. Hence, little is known about which phenolic plays a leading role in the anti-proliferative effect on cancer. Thus, more investigations are still needed to understand the anti-proliferative effect of individual phenolic from sorghum grain.

### 3.4. Anti-Diabetic Effect

Diabetes is one of the most challenging chronic diseases worldwide. The insulin resistance and pancreatic b-cell dysfunction result in the hyperglycemia and abnormal carbohydrate metabolism, further leading to type 2 diabetes (T2D). Sorghum phenolic extracts have been found to effectively inhibit diabetes, through reducing serum glucose, total cholesterol, and triglycerides [6,57,58]. This section will focus on the anti-diabetic effect of sorghum phenolic extracts in vitro and in vivo.

Chung et al. [57] found that phenolic extracts of Hwanggeumchal sorghum (250 and 500 mg/kg for 14 days) could significantly reduce the serum glucose, total cholesterol, triglycerides, urea, uric acid, creatinine, aspartate amino transferase, and alanine amino transferase in streptozotocin-induced diabetic rats. In addition, phenolic extracts of Hwanggeumchal sorghum at 250 mg/kg also resulted in an increase in serum insulin in diabetic rats, but not in normal rats [57]. However, the mechanism of the anti-diabetic effect of sorghum phenolics was not discussed. Phenolic extracts of Hwanggeumchal sorghum (0.5% and 1% addition to dietary intake) given to high fat diet-fed rats resulted in a significant reduction in perirenal fat, total and low-density lipoprotein cholesterol (LDL-cholesterol), triglycerides, and glucose [59]. The hypoglycemic effect of sorghum extracts was considered to be associated with the regulation of PPAR-g-mediated metabolism in rats. The anti-diabetic effect of sorghum extract was also evaluated in diabetic rats induced by streptozotocin, and results showed sorghum extracts (0.4 and 0.6 g/kg) decreased the expression of phosphoenolpyruvate carboxykinase and the phosphor-p38/p38 ratio, but did not affect the glucose transporter 4 translocation and the phosphor-Akt/Akt ratio [60]. Similarly, Wu et al. [58] also reported that feeding sorghum red pigments (200 mg/kg body weight) to diabetic mice induced by glucose reversed glucose tolerance and serum levels of triglycerides, total and LDL-cholesterol, and ameliorated lipid metabolism. In addition, the feeding of sorghum red pigments (200 mg/kg body weight) to diabetic mice reduced body weight by 26.5%, compared to the diabetic mice [58]. However, the mechanism of the anti-diabetic effect of sorghum phenolics was not shown.

In addition to the phenolic extracts from sorghum grain, sorghum flour has also been shown to modulate adiposity and inflammation in obese rats fed with a high fat diet. Tested diets (replacement of 50% cellulose and 100% of corn starch by sorghum flour, and replacement of 100% cellulose and 100% of corn starch by sorghum flour in obese diet) lowered the percentage of adiposity, fatty acid synthase gene expression, TNF-α, blood levels of glucose, and adipocyte hypertrophy in rats [6].

In addition, polyphenolics and anthocyanins have been reported to inhibit the starch digestive enzymes, such as α-amylase and α-glucosidase, thus retarding starch digestibility and lowering the value of glucose index (GI), which is also considered to possess the anti-diabetic effect [28,47]. The IC_50_ values of the inhibitory effect of proanthocyanidins from Sumac sorghum and black sorghum bran phenolic extracts on α-amylase were reported to be 1.4 and 11.4 mg/mL, respectively [51]. The effect of red sorghum phenolic extract on pancreatic lipase inhibition, α-amylase activity, and α-glucosidase inhibitory activity was studied by Irondi et al. [61], and they found that IC_50_ values were 12.72 ± 1.13, 16.93 ± 1.08, and 10.78 ± 0.63 mg/mL, respectively. IC_50_ values of brown sorghum genotypes (SOR 01, SOR 03, SOR 08, SOR 11, SOR 17, SOR 21, SOR 24, SOR 33) on α-glucosidase and α-amylase inhibition were reported to be 14.7 to 61.0 mg/mL and 10.6 to 852.6 mg/mL, respectively [28]. The anti-diabetic effect of sorghum phenolic extracts is summarized in Table 5.

To date, studies have shown the anti-diabetic effect of sorghum phenolics, including the reduction in serum glucose, decrease in total cholesterol and triglycerides, and inhibition of α-glucosidase and α-amylase activity. However, the underlying mechanisms of the inhibitory effect of sorghum phenolics on diabetes have not been fully studied. Therefore, more research is warranted for further investigation of the role of sorghum phenolics in diabetes inhibition.

### 3.5. Anti-Atherogenic Effect

Atherosclerosis is a major factor in the development of coronary heart disease. Blood vessel walls turn thicker in the development of atherosclerotic lesions, thus affecting blood circulation. Many risk factors are involved in the induction of heart disease, such as hypercholesterolemia, hypertension, cigarette smoking, and diabetes [62]. Moreover, hypercholesterolemia is associated with an increased level of LDL-cholesterol. Hence, hypercholesterolemia is a major risk factor for the development of cardiovascular disease. An increase in total cholesterol, triglycerides, and LDL-cholesterol are all considered to be associated with an increased risk of atherosclerosis and cardiovascular diseases, whereas high-density lipoprotein cholesterol (HDL-lipoprotein) is considered to be associated with the reduced risk of atherosclerosis and cardiovascular disease [62].

The anti-atherogenic effect of sorghum on high fat diet-fed mice was studied by Shen et al. [22], and they found that the diet containing sorghum reduced serum cholesterol by 24.47% and triglyceride by 32.72%, and increased HDL-cholesterol by 27.27% compared to the high fat diet group. In addition, the sorghum feed also increased SOD and GPx activities in the serum, compared to high fat diet mice [22]. A high fat diet might result in the generation of ROS, which can be controlled through enzymatic defense mechanisms, such as SOD, GPx, and CAT. Phenolics in sorghum possess a strong antioxidant capacity, thus increasing the activities of enzymes, SOD and GSH-Px, for reducing the oxidative effect. To date, little is known about the anti-atherogenic effect of sorghum phenolic extracts, as well as the underlying mechanism both in vitro and in vivo. Therefore, more studies are needed to better understand the anti-atherogenic effect of sorghum phenolics.

## 4. Effect of Processing on Phenolic Compounds in Sorghum

Sorghum grain is a good alternative to cereal grains, due to it containing nutritional and health-promoting factors. However, tannins, known as bitter-tasting compounds, have negative effects on human consumption, although they can provide biological functions for improving human health. So far, to our knowledge, sorghum grain is thermally cooked, such as through boiling, cooking, nixtamalization (alkaline cooking), extrusion, roasting, and steaming prior to human consumption. Tannins and polyphenols are considered as anti-nutritional factors which affect the digestion of starch and protein. Thermal processing could potentially reduce the content of tannins and phenolic compounds [63,64,65]. Therefore, the biological functions of phenolic compounds will also be affected by thermal processing, due to heat sensitivity. Thus, this section will focus on the effect of thermal processing on phenolic compounds in sorghum varieties. In addition, the current research gap and future research topics will also be discussed.

The reduced content of tannins in sorghum cultivars (Wadakar, low b-glucan type II non-tannin sorghum; Tabat-C, low b-glucan type I non-tannin sorghum; new in bread line Tabat-NL, high b-glucan type I non-tannin sorghum) has been reported through cooking (20 min) and boiling also decreased the content of phytate and polyphenols [64]. In addition, Hamad et al. [64] also reported that boiling resulted in an increase in digested starch, rapid digestible starch, hydrolysis index, and estimated GI, indicating that boiling eliminated anti-nutritional factors, such as tannins and polyphenols, to further improve starch degradation [64]. Luzardo-Ocampo et al. [66] reported that cooking and nixtamalization (10 g Ca(OH)_2_/kg flour, 94 °C for 40 min) significantly reduced the content of condensed tannins, total phenolics, and flavonoids in the digestible fraction white sorghum (Tortillas y Pan), whereas it increased the content of total phenolics and flavonoids in the digestible fraction of red sorghum (Niquel) compared to the raw sorghum [66]. The variance in the content of condensed tannins, total phenolics, and flavonoids in white and red sorghums was believed to result from the different degree of cooking and nixtamalization in the undigested form, mouth, stomach, digestible fraction, and non-digestible fraction [66].

Pressured steam cooking and air-dried flaking also resulted in the decrease in content of phytate and tannins, from 69.87% to 93.73% and 19.49% to 46.05%, respectively, in sorghum varieties of IS8237C, Liberty, and Alpha [67]. Xiong et al. [64] showed that steaming (100 °C for 50 min) and roasting (150 °C for 60 min) increased the content of total phenolics, total flavonoids, and condensed tannins to a different extent in non-tannin white color sorghum (Liberty). Steaming and roasting can damage the cellular structure of cereal grains, thus releasing the bound phenolic compounds, and increasing the content of phenolics after thermal processing [68,69]. Extrusion cooking has also been shown to decrease the content of total phenols and total flavonoids of sorghum genotypes SC319, B.DLO357, and SC391 [70].

To date, more studies have been focused on the effect of thermal processing on nutrients digestion in sorghum grains, rather than on the phenolics regarding the content and biological functions after thermal processing. Therefore, more research is recommended to better understand the effect of phenolics in sorghum grains, with regards to the content, profile, and biological activities through thermal treatments.

## 5. Conclusions and Outlook

This review discussed the phenolic compounds in sorghum grain, in terms of the extraction method, profile, and biological functions both in vitro and in vivo. Representative phenolic compounds in sorghum are ferulic acid, caffeic acid, gallic acid, luteolin, apigenin, 3-DXA, and others. Bioactive phenolic compounds possess many biological functions, such as an antioxidant activity, anti-inflammatory effect, anti-proliferative effect, anti-diabetic, and anti-atherogenic effects. However, the underlying mechanisms regarding the inhibitory effect of sorghum phenolics on inflammation, diabetes, and atherosclerosis remain unclear. Therefore, more studies are warranted for a better understanding of the molecular mechanisms involved in the inhibitory effect of sorghum phenolics on inflammation, diabetes, and atherosclerosis.

To date, sorghum grain has been served as a cereal grain alternative, or has replaced wheat or other cereal grains in innovative bakery products for human consumption. The thermal processing could potentially reduce the content of tannins and phytate, which are the anti-nutritional factors for improving nutrient digestion. However, the loss of biological functions of phenolics and tannins in sorghum has also been reported. Although steaming and roasting processes increased the content of total phenolics and flavonoids due to the release of bound phenolics, most studies showed a reduction in phenolics in sorghum grains after thermal treatment. Therefore, moderate processing is urgently required for maintaining the content of phenolics and their biological activities, and for reducing the anti-nutritional factors in sorghum grains to improve nutrient digestion.

## Figures and Tables

**Table 1:** Total phenolics content (TPC), total flavonoids content (TFC), total anthocyanins content (TAC) of sorghum varieties associated with pigmented pericarps.

Sorghum Source	TPC (mg/g)	TFC (mg/g)	TAC (mg/g)	Reference
White pericarp	0.24–34.78 GAE	0.06–0.38 RE	0.02 CCE; 0.09 GAE	[2,21,22]
Yellow pericarp	-	-	Bran: 0.26–0.81 AE; Flour: 0.10–0.35 AE	[3]
Black pericarp	4.13–11.50 GAE	0–0.20 RE	3.02 GAE, 0.18 CCE	[2,21]
Brown pericarp	3.58 GAE; 1.74 FAE	1.39 CE	5.55 GAE;	[21]
Red pericarp	0.66–47.86 GAE	0–0.60 RE	0.41–0.60 GAE; 2.66–8.93 CCE	[21,22]

GAE: gallic acid equivalents; RE: rutin equivalents; CCE: cyanidin chloride equivalents; FAE: ferulic acid equivalents; CE: catechin equivalents; AE: apigenidin equivalents.

**Table 2:** Antioxidant activities of phenolic extracts of sorghum grains.

Sorghum Source	Bioactive Extracts	Antioxidant Activity	Reference
Hongyingzi, Hongzhenzhu, Dongbei sorghum, Jiangsu sorghum, Jiliang 2 sorghum, Longza 11, black grain sorghum, white Longmi sorghum.	Caffeic acid, *p*-coumaric acid, ferulic acid, protocatechuic acid, luteolindin, apigeninidin, luteolin, apigenin, taxifolin, naringenin.	Antioxidant activities against DPPH and FRAP assays.	[2]
Tannin-containing sorghum varieties (Sumac, Hi-Tannin, Seredo, CR 35:5 × 2), non-tannin varieties (white variety, KARI-Mtama, red variety, ICSV-III), Mizzou, Tx430.	Condensed tannins, 3-DXA, phenolics.	Induced phase II detoxifying enzymes; anti-proliferative effect on esophageal, OE33, colon cancer cells.	[4]
Liberty, Mr-Buster, Cracker, IS131C, Shawaya Short Black 1.	Phenolic extracts.	Antioxidant activities against DPPH and FRAP assays; Anti-proliferative effect on Caco-2 cells.	[15]
Tx3362, Shawaya Black, Black PI Tall, Hyb 107, Hyb 115, Hyb 116, Hyb 117, Hyb 118.	Total phenolics, condensed tannins, flavan-4-ols, 3-DXA.	Antioxidant activities against DPPH and ABTS assays.	[5]

**Table 3:** Anti-inflammatory effect of phenolic extracts of sorghum grains.

Sorghum Source	Bioactive Extracts	Anti-Inflammatory Effect	Reference
Red sorghum.	Phenolics, flavonoids, anthocyanins.	Antioxidant activities against DPPH, FRAP, superoxide radical scavenging, hydroxyl radical scavenging assays, metal chelating, hydrogen peroxide.	[23]
Tong Za 117, Tong Za 141, Tong Za 142, Tong Za 143, Chi Za 109, Chi Za 101.	Ferulic acid, p-coumaric acid, caffeic acid, 3,4-dihydroxybenzoic acid, luteolinidin, apigeninidin, 5-methoxyluteolinidin, 7-methoxy apigeninidin.	Antioxidant activities against DPPH and ABTS assays; inhibitory effect on IL-6 and IL-1β.	[37]
Sumac, Mycogen 726, black sorghum, white sorghums.	Total phenolic extracts.	Inhibitory effect on IL-1β and TNF-α.	[38]
SC84MX, SC84KS, PI57048.	Phenolics, flavonoids, tannins, 3-DXA, anthocyanins.	Antioxidant activities against DPPH, ORAC and nitric oxide assays; inhibited cellular production of NO, IL-6, ROS.	[39]
PUI570481	Polyphenol extracts.	Inhibitory effect on IL-6 and TNF-α.	[42]
1-Terral REV 9924, 2-Pioneer 84P8D, 3-Dekalb DK-54-00, 4-FFR353, 5-DynaGro DG765B, 6-Pioneer 83P99, 7-Dekalb DK-51-01, 8-Terral REV 9782, 9-Terral REV 9562, 10-Terral REV9883.	Naringenin, eriodicytol, apigenin, luteolin, apigeninidin, luteolinidin.	Antioxidant activities against DPPH and NO assays; Inhibitory effect on OVCA cells.	[43]
White sorghum, red sorghum.	Gallic acid, protocatechuic acid, chlorogenic acid, caffeic acid, luteolinidin, apigeninidin, p-coumaric acid, flavanols, quercetin, hydroxycinnamic acid, 5-methoxy luteolinidin, 7-methoxy-luteolinidin, 5,7-dimethoxy-luteolinidin, 7-methoxy-apigeninidin, 5,7-dimethoxy-apigeninidin.	Antioxidant activities against ORAC and nitric oxide assays; inhibited cellular production of NO, IL-6, ROS.	[44]

**Table 4:** Anti-proliferative effect of phenolic extracts of sorghum grains.

Sorghum Source	Bioactive Extracts	Anti-Proliferative Effect	Reference
Black sorghum varieties (Macia, Sumac, PI152653, PI152687, PI193073, PI1329694, PI1559733, PI1559855, PI1568282, PI1570366, PI1570481, PI1570484, PI1570819, PI1570889, PI1570993).	Total phenolic extracts.	Anti-proliferative effect on HepG2 and Caco-2 cells: induction G1/S cell cycle arrest, activation of p53.	[48]
Red sorghum	3-DXA extracts.	Inhibitory effect on MCF7 cancer cells through up-regulating p53 and down-regulating Bcl-2 genes.	[52]
Dale, M81E	Vanillic acid, p-coumaric acid, ferulic acid, caffeic acid, apigeninidin, luteolinidin, malvidin-3-*O*-glucoside, apigenin, luteolin, trans-resveratrol, luteoferol.	Inhibitory effect on HCT116 and colon cancer stem cells through activating p53 gene.	[53]
Hwanggeumchal sorghum.	Total polyphenol extracts.	Anti-proliferative effect on MDA-MB 231 and MC7 cells: down-regulating VEGF, VEGF-R2, cyclin D, cyclin E, pRb and up-regulating p53.	[55]
TX430, Sumac.	Total phenolic extracts.	Anti-proliferative effect on HepG2 and HCT15 cells.	[56]

**Table 5:** Anti-diabetic and anti-atherogenic effect of phenolic extracts of sorghum grains.

Sorghum Source	Bioactive Extracts	Anti-Diabetic and Anti-Atherogenic Effects	Reference
Brown sorghum varieties (SOR 01, SOR 03, SOR 08, SOR 11, SOR 17, SOR 21, SOR 24, SOR 33)	Gallic acid, chlorogenic acid, caffeic acid, ellagic acid, p-coumaric acid, quercetin, luteolin, apigenin.	Inhibitory effect on α-amylase and α-glucosidase activities.	[28]
Hwanggeumchal sorghum.	Phenolic extracts.	Reduced the serum glucose, total cholesterol, triglycerides, urea, uric acid, creatinine.	[57]
KNICS-579	Polyphenol extracts.	Reduced the concentration of triglycerides, total LDL-cholesterol and glucose.	[60]
Red sorghum	Total phenolic extracts.	Antioxidant activity against ABTS, DPPH, FRAP assays; Inhibitory effect on pancreatic lipase, α-amylase and α-glucosidase activities.	[61]

## Data Availability

Not applicable.
